# Pax8 plays a pivotal role in regulation of cardiomyocyte growth and senescence

**DOI:** 10.1111/jcmm.12779

**Published:** 2016-01-19

**Authors:** Yihao Wu, Xi Zhou, Xiaoyan Huang, Quan Xia, Zhe Chen, Xingwei Zhang, Deye Yang, Yong‐jian Geng

**Affiliations:** ^1^ Division of Cardiology The First Affiliated Hospital of Wenzhou Medical University Wenzhou China; ^2^ Division of Cardiology The Affiliated Hospital of Hangzhou Normal University Hangzhou China; ^3^ The University of Texas School of Medicine at Houston Houston TX USA

**Keywords:** Pax8, ventricular septal defect, ALK3, senescence, apoptosis

## Abstract

Congenital heart disease (CHD) is a worldwide health problem, particularly in young populations. In spite of the advancement and progress in medical research and technology, the underlying causative factors and mechanisms of CHD still remain unclear. Bone morphogenetic protein receptor IA (ALK3) mediates the development of ventricular septal defect (VSD). We have recently found that paired box gene 8 (Pax8) may be the downstream molecule of ALK3. Paired box gene 8 plays an essential role in VSD, and apoptosis and proliferation imbalance leads to septal dysplasia. Recent studies have also disclosed that cellular senescence also participates in embryonic development. Whether programmed senescence exists in cardiac organogenesis has not ever been reported. We hypothesized that together with various biological processes, such as apoptosis, enhanced cellular senescence may occur actively in the development of Pax8 null mice murine hearts. In H9C2 myogenic cells, Pax8 overexpression can rescue caspase‐dependent apoptosis induced by ALK3 silencing. Senescent cells and senescence‐associated mediators in Pax8 knockout hearts increased compared with the wild‐type ones in an age‐dependent manner. These results suggest that Pax8 maybe the downstream molecule of ALK3, it mediates the murine heart development perhaps *via* cellular senescence, which may serve as a mechanism that compensates for the cell loss *via* apoptosis in heart development.

## Introduction

Congenital heart disease (CHD) is one of the fastest growing problems in cardiology 1. Regulation of cardiac development depends on spatial and temporal expression of signalling molecules and other regulators. Classical signalling pathways participates in ventricular septum morphogenesis, such as bone morphogenetic proteins (BMPs), Wnt signalling, fibroblast growth factor, and the latest microRNA and long non‐coding RNA 2, 3, 4, 5. Bone morphogenetic proteins belong to the transforming growth factor‐β (TGF‐β) superfamily, which regulates a broad spectrum of developmental events, including cardiac cushions 6. As an important BMP receptor, BMP receptor IA (ALK3) is crucial for cardiac myocyte differentiation and heart development 7, 8. Cardiac‐specific ALK3 gene deletion (ALK3^−/−^) mice are lethal at mid‐gestation with defects in the endocardial cushion, trabeculae and interventricular septum 9.Our previous study has revealed that the paired box gene 8 (Pax8) expression is down‐regulated by 7.1‐fold in cardiac‐specific ALK3^F/−^ mice filtered by high‐throughput biochip 10. The function of Pax8 in heart development has never been studied previously, we wonder that whether Pax8 is the downstream molecule of ALK3 in ventricular septum morphogenesis. Our previous study found that Pax8 null mice presented the ventricular septum defect and increased apoptotic myocytes 11. Paired box gene 8 may regulate ventricular septal defect (VSD) by affecting the trade‐off between apoptosis and proliferation.

Apoptosis has a critical function in tissue development and maturation, as a form of programmed cell death 12. One of the potential mechanisms for septum dysplasia has been attributed to apoptosis 13. Recent report has shown that cells may undergo programmed senescence during mammalian embryonic development 14, 15. Thus, apoptosis and senescence may serve as two potential mechanisms of heart dysplasia. Abnormal cardiac development in Pax8 null mice hearts may result from irregulated apoptosis and senescence. In general, senescence is defined as cell‐cycle arrest, first described by Hayflick in 1965 16. Together with apoptosis, senescence is a biological response to risk factors, which activate both apoptosis and senescence. The two bioprocesses may share common triggers, such as oncogenic stress or genomic instability, common with regulators, particularly p53 17, 18. To understand the mechanism underlying the senescence and apoptosis of cardiac cells during the development of Pax8 null hearts, we investigated whether Pax8 could rescue apoptosis induced by ALK3and whether cell senescence contributes to defects in the development in Pax8 null hearts.

## Materials and methods

### Cell cultures

Animal experiments have been approved by the Animals Welfare Committee of Wenzhou Medical University. All the animals received human care. H9C2 cells were obtained from the Cell Bank of Chinese Academy of Sciences (Shanghai, China). Cells were grown as a monolayer culture in high glucose DMEM (Gibco, Carlsbad, CA, USA) supplemented with 10% foetal bovine serum (FBS; Gibco). Mouse myocardial cells were prepared from neonatal (born within 3 days) C57/B6J mice. Heart tissues were repeatedly digested by collagenase II (Roche, Basel, Switzerland) in 37°C water bath. All myocytes were filtrated in a 200 mesh strainer. Cells remained in suspension were seeded in 6‐well plates at a density of 1 × 10^5^ cells per well with a differential plating step of 1 hr 19. The medium used was consisted of high glucose DMEM, 20% FBS, 1% penicillin and 1% streptomycin. The cultures were maintained in a humidified incubator with 5% CO_2_ at 37°C.

### Construction of Pax8/pcDNA3.1 and ALK3 shRNA/pGPU6 expression vectors

The cloning vector pCMV sport6 containing the full length rat Pax8 gene was purchased from ATCC (Manassas, USA). The full length Pax8 gene was excised by KpnI and NotI restriction enzymes, and inserted into the expression vector pcDNA3.1 (+) (Invitrogen, Carlsbad, CA, USA). The recombinant plasmid Pax8/pcDNA3.1 was confirmed by PCR, restriction endonuclease digestion and sequencing. Basing on the published sequence of ALK3, we made four short hairpin RNAs (shRNAs) for ALK3, namely ALK3shRNA‐1, ALK3 shRNA‐2, ALK3shRNA‐3, ALK3shRNA‐4 (Table 1), and inserted them into pGPU6 vector (Invitrogen). The sequences of Pax8 small interfering RNA (siRNAs) are shown in Table S2. ALK3 shRNAs in pGPU6 were confirmed by restriction endonuclease digesting and sequencing.

**Table 1:** The target sequence for ALK3

Name sequence	
ALK3 shRNA‐1	
Sense	5‐CACCGGAGAAACCACGTTAACTTCTTTCAAGAGAAGAAGTTAACGTGGTTTCTCCTTTTTTG‐3
Anti‐sense	5‐GATCCAAAAAAGGAGAAACCACGTTAACTTCTTCTCTTGAAAGAAGTTAACGTGGTTTCTCC‐3
ALK3 shRNA‐2	
Sense	5‐CACCGCTAGCTGGTTTAGAGAAACATTCAAGAGATGTTTCTCTAAACCAGCTAGCTTTTTTG‐3
Anti‐sense	5‐GATCCAAAAAAGCTAGCTGGTTTAGAGAAACATCTCTTGAATGTTTCTCTAAACCAGCTAGC‐3
ALK3 shRNA‐3	
Sense	5‐CACCGGACTCAGCTGTATTTGATTATTCAAGAGATAATCAAATACAGCTGAGTCCTTTTTTG‐3
Anti‐sense	5‐GATCCAAAAAAGGACTCAGCTGTATTTGATTATCTCTTGAATAATCAAATACAGCTGAGTCC‐3
ALK3 shRNA‐4	
Sense	5‐CACCGCCTAGCTGTTAAATTCAACATTCAAGAGATGTTGAATTTAACAGCTAGGCTTTTTTG‐3
Anti‐sense	5‐GATCCAAAAAAGCCTAGCTGTTAAATTCAACATCTCTTGAATGTTGAATTTAACAGCTAGGC‐3
Negative control	
Sense	5‐CACCGTTCTCCGAACGTGTCACGTCAAGAGATTACGTGACACGTTCGGAGAATTTTTTG‐3
Anti‐sense	5‐GATCCAAAAAATTCTCCGAACGTGTCACGTAATCTCTTGACGTGACACGTTCGGAGAAC‐3

### Western blot analysis

Total protein concentration was quantified by bicinchoninic acid assay protein assay kit (Pierce, Rockford, AL, USA). Protein lysates (50–80 μg) were fractionated on 10% SDS‐PAGE after heating for 5 min. at 100°C and transferred onto polyvinylidene difluoride membranes. After blocking with 5% non‐fat dry milk, the membranes were incubated overnight at 4°C with primary antibody specific for Pax8 (1:100 dilution; Abcam, Cambridge, UK), cleaved caspase‐3 antibody (1:800; Abcam), cleaved caspase9 antibody (1:500; Abcam), p21 (1:200; Santa Cruz, Dallas, TX, USA), p53 (1:500; Abcam), and Hp1γ (1:1000; Cell Signaling, Boston, MA, USA). Then the membranes were incubated for 1 hr with a secondary antibody conjugated to horseradish peroxidase (1:2000 dilution; Abcam). GAPDH or β‐actin was used as internal control. The bands were finally visualized by an enhanced chemiluminescence detection system. The relative intensity of the bands of interest was analysed by Quantity One software.

### Cell proliferation assays

Cells (6 × 10^3^ cells/well) were seeded in a 96‐well microtiter culture plate. Cell proliferation was determined by CCK‐8 kit (Dojindo, Kyushu, Japan) according to the instructions of the manufacturer. After incubation for 48 hrs, CCK‐8 solution was added to cells. Cells were incubated at 37°C for 60–90 min. The optical density of each well was examined at 450 nm using a microplate reader (ELX800; Bio‐Tek, Highland Park, FL, USA).

### Immunofluorescence

Cardiomyocytes and heart cryosections were fixed with paraformaldehyde, and permeabilized with 0.5% TritonX‐100 in PBS. The following treatment with goat serum was used to block the non‐specific binding sites for 1 hr at room temperature. The cultured cells were incubated with α‐myosin heavy chain (α‐MHC) mouse monoclonal antibody (1:100; Abcam), and then with tetramethylrhodamine‐conjugated second antibody (1:1000; Jackson, Bar Harbor, ME, USA). Slices were incubated with Hp1γ (1:600) and donkey anti‐rabbit IgG H&L (1:500; Abcam). The nuclei were stained with 4′,6‐diamidino‐2‐phenylindole (DAPI). Staining was subsequently observed by a fluorescence microscope.

### Pax8‐lentiviral vector production

The mouse Pax8 target gene segment (ATCC) was amplified by PCR, and amplified Pax8 cDNA was ligated into the PLVEFA1α/red fluorescent protein (RFP; LV7) vector (GenePharma, Shanghai, China) after purification, digestion, transformation, and sequencing. The recombination shuttle vector was cotransfected with packaging plasmids mixed with pGag/Pol (containing the gag/pol), pRev (encoding the Rev protein) and PVSV‐G plasmids (encoding envelope protein) into 293T cells (The Cell Bank of Chinese Academy of Sciences). Viral particles were collected after transfection for 72 hrs. The re‐suspended vector pellet was centrifuged and tittered.

### Real‐time PCR

RNA extraction and cDNA synthesis were performed as described above. Their purity and concentration were detected using ultraviolet spectrophotometer (NanoDrop‐1000; Thermo, Fremont, CA, USA), whereas their integrity was detected by agarose gel electrophoresis with a gel‐imaging system (Pharmacia Biotech, Shinjuku‐ku, Tokyo, Japan). Real‐time PCR measurements were made using the Power SYBR Green PCR Master Mix (ABI, New York, NY, USA) with output to a computer‐based acquisition system (ABI 7900 sequence detector). Reaction condition: the first denaturation for 5 min. at 95°C, 95°C for 15 sec., 64°C for 34 sec., for a total of 40 cycles. All samples repeated the three sub‐wells. Primer sequences are shown in Table S1.

### Flow cytometry

Neonatal cardiomyocyte apoptosis was measured using an Annexin V‐FITC/PI Assay Kit (Invirtrogen) according to the instructions of the manufacturers. The percentages of apoptotic cells were analysed by fluorescence‐activated cell sorter (BD Biosciences, San Jose, CA, USA).

### SA‐β‐gal staining

Heart was freshly isolated from E18 embryos and P14 mice, embedded in Tissue Freezing Medium (Leica Jung, Solms, Germany), frozen, and serially sectioned. Slides were fixed in fixation buffer, and according to the manuscripts of Senescence Cells Histochemical Staining Kit (Sigma‐Aldrich St. Louis, MO, USA).

### Immunohistochemistry

Fresh hearts at embryo of day 18 (E18) and postnatal day 14 (P14) were fixed in 4% paraformaldehyde for 18 hrs, dehydrated, embedded in paraffin, cut to 3 μm, and dewaxed by routine. Subsequently, 0.3% H_2_O_2_ was used to inactivate endogenous peroxidases. Antigen retrieval was boiled for 3 min. in citrate buffer (pH 6.0) in a pressure cooker and permeabilized with 0.5% TritonX‐100 for 15 min. The slides were blocked using goat serum for 30 min. at room temperature, and incubated with primary antibodies (p21 was diluted to 1:100, p53 was diluted to 1:150) for 1 hr. The secondary antibody was incubated at 37°C and stained with 3,3′‐diaminobenzidine DAB staining for 3 min. The slides were counterstained by haematoxylin, dehydrated and sealed with rhamsan gum. Images were captured using a Nikon Ti‐E inverted fluorescence microscope (Midori, Otawara, Tochigi, Japan).

### Statistical analysis

All the quantitative data were analysed using Spss17 software. The normality test (Kolmogorov‐Smirnov test) and homogeneity test of variance (Levene test), one‐way anova, and Bonferroni test were performed based on the experimental design. Measurements are presented as means ± S.D. When *P* < 0.05 the difference between means was considered statistically significant.

## Results

### Expression of Pax8 and ALK3 in H9C2 cells

**Figure 1:**
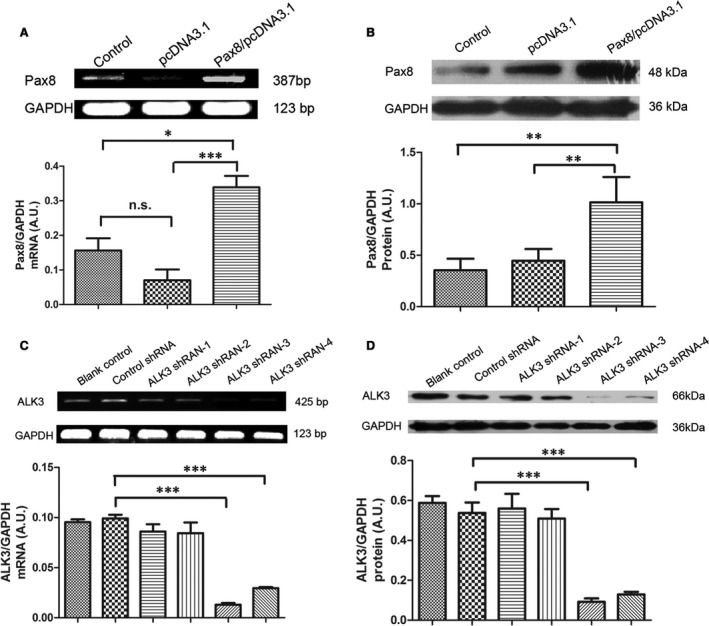
Pax8 overexpression and ALK3 down‐expression in H9C2 cells. (**A**) Pax8 mRNA expression was analysed using semi‐quantitative Real‐time PCR. Pax8/pcDNA3.1 was transfected in cells for 24 hrs. The pcDNA3.1 alone was used as negative control. (**B**) Pax8 protein level was quantified by Western blot analysis. (**C**) ALK3 mRNA level in H9C2 cells. (**D**) ALK3 protein level in H9C2 cells transfected with ALK3 shRNAs. Note that ALK3shRNA‐3 and ALK3shRNA‐4 significantly silenced ALK3 protein expression. Data are presented as mean ± S.D. *, *P* < 0.05; **, *P* < 0.01;***, *P* < 0.001.

The expression vector construction was confirmed by the restriction sites of the expression plasmids *via* PCR and sequencing. These cDNA fragments were subcloned into pGPU6 vector and also confirmed by restriction mapping and sequencing. Semi‐quantitative RT‐PCR and Western blot analysis showed increased Pax8 expression in H9C2 cells transfected with Pax8/pcDNA3.1 after 48 hrs (Fig. 1A and B). Paired box gene 8 mRNA expression apparently increased in cells transfected with Pax8/pcDNA3.1 compared with cells transfected with pcDNA3.1 plasmid alone and un‐transfected cells. In parallel to the mRNA expression, a large increase in Pax8 protein level was observed in cells transfected with Pax8/pcDNA3.1. In H9C2 cells treated with only ALK3 shRNA‐3 and ALK3 shRNA‐4, but not with ALK3 shRNA‐1 and ALK3 shRNA‐2, expressed lower levels of ALK3 mRNA and protein (Fig. 1C and D). We have used the ALK3 shRNA‐3 in all the following experiments.

### Pax8 rescues proliferation inhibition induced by ALK3

**Figure 2:**
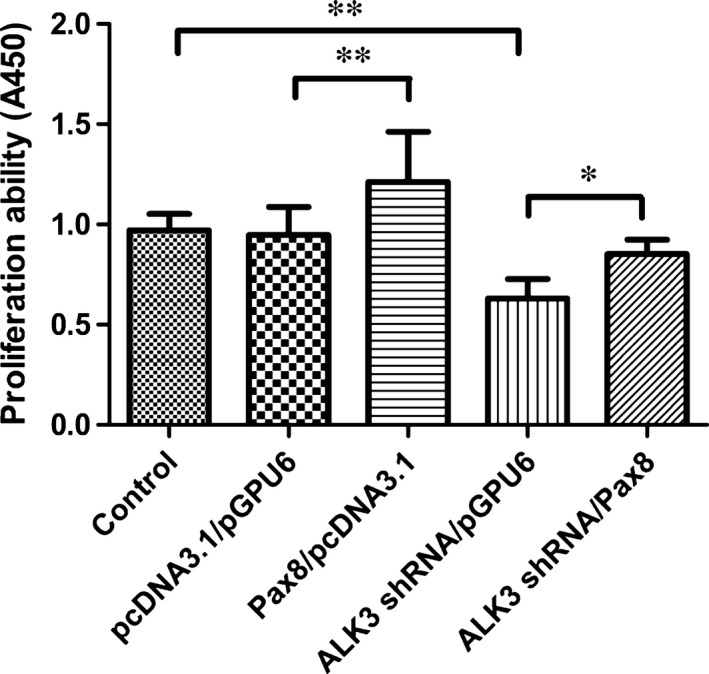
Pax8 overexpression rescued proliferation inhibition by ALK3 silence in H9C2 cells. Proliferation was detected by CCK8 kit by using microplate reader at wavelength A450 nm. The pcDNA3.1 and pGPU6 were vectors of Pax8 and ALK3 respectively. Data are presented as mean ± S.D. *, *P* < 0.05; **, *P* < 0.01.

As shown in Figure 2, H9C2 cells exposed to Pax8/pcDNA3.1 had an increased rate of proliferation compared with that of cells un‐transfected or transfected with pcDNA3.1/pGPU6 alone. By contrast, transfection with ALK3 shRNA‐3 inhibited the proliferation of H9C2 cells by about 50% compared with un‐transfection or transfection with pcDNA3.1/pGPU6. Interestingly, the inhibitory effect of shRNA‐mediated ALK3 silencing could be partly compensated by con‐transfecting with the Pax8 gene. Thus, we concluded that Pax8 rescued the proliferation inhibition induced by ALK3 silencing.

### Effect of Pax8 on ALK3‐induced apoptosis in H9C2 cells

**Figure 3:**
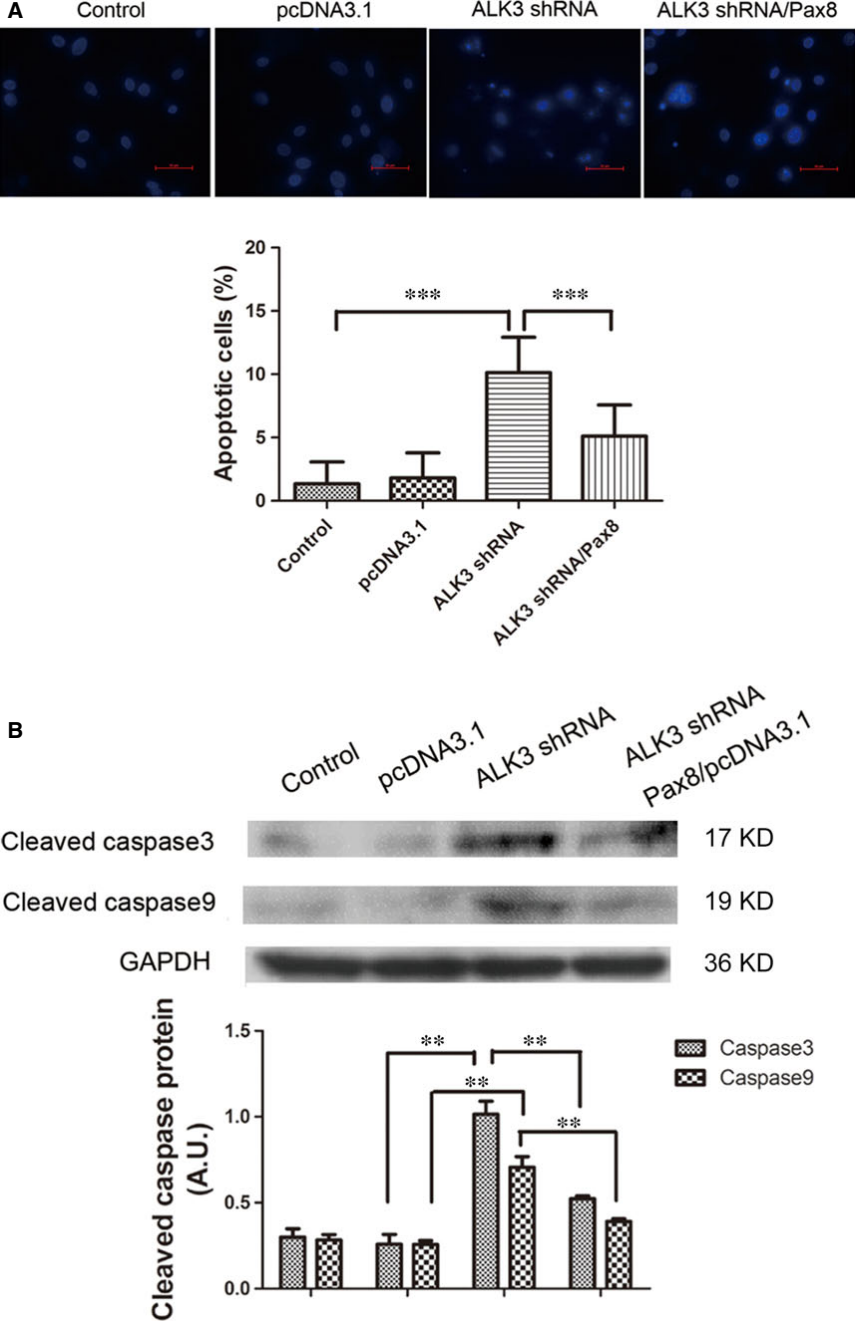
Pax8 rescued apoptosis induced by ALK3 silence in H9C2 cells. (**A**) Apoptosis was observed by DAPI staining by using a fluorescence microscopy. Positive apoptotic cells were indicated by bright blue staining. Ten different visual fields were randomly chosen to count positive cells. The percentage of positive cells relative to the total cells was considered as the rate of apoptosis in each visual field. (**B**) The H9C2 cells performed as a control group, negative control were transfected with pcDNA3.1and pGPU6 vectors. The ALK3 silence group was transfected with ALK3 shRNA/pGPU6. Treating group was pre‐treated with Pax8/pcDNA3.1 24 hrs earlier and ALK3 shRNA/pGPU6. Data are presented as mean ± S.D. **, *P* < 0.01. ***, *P* < 0.001 (magnification: ×400).

The percentage of karyopyknosis cells by DAPI staining was observed by fluorescence microscopy. Positive apoptotic cells showed bright blue fluorescence, chromatin concentration and nucleus fragmentation. When pre‐treated with Pax8/pcDNA3.1, there was a reduction in DAPI‐positive cells relative to total cells, as counted randomly in 10 different visual fields from several different experiments. Moreover, the apoptosis of H9C2 cells induced by ALK3 silencing was inhibited by Pax8 overexpression (Fig. 3A). In addition, analysis of apoptosis‐associated activities of caspase‐3 and 9 by Western blot analysis showed that transfection with ALK3 shRNA‐3 to silence ALK3 expression induced a higher amount of activated caspase‐3 protein level compared with un‐transfection or transfection with pcDNA3.1/pGPU6 only. Overexpression of Pax8 could significantly reduce caspase‐3 activity induced by ALK3 gene silencing (Fig. 3B). Similarly, activated caspase‐9 increased in cells after ALK3 gene silencing, whereas the activity was reduced by Pax8 overexpression.

We found that ALK3 induced apoptosis *via* caspase‐dependent way, whereas Pax8, partly alleviated the caspase‐dependent. Moreover, we found that activated caspase‐3 increased with Pax8 knockdown evoked by ALK3 silencing (Fig. S1).

### Anti‐apoptotic effect of Pax8 in primary myocardial cells

**Figure 4:**
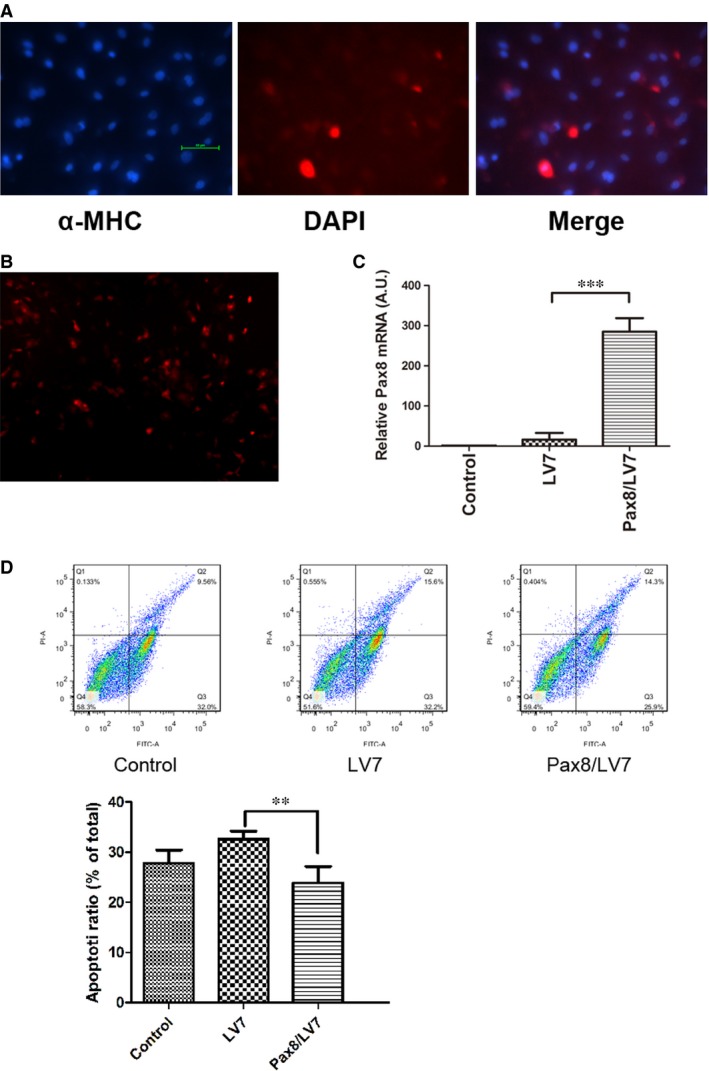
Pax8 rescued apoptosis in neonatal mice cardiomyocytes. (**A**) Identification of primary cardiomyocytes. The α‐MHC staining specifically shows orange red staining in plasma of cardiomyocytes, whereas blue spots are observed in DAPI‐stained nucleus. (**B**) Pax8 overexpression vectors were transfected in myocytes with the photo taken from an inverted fluorescence microscope. (**C**) Real‐time PCR analysis of Pax8 mRNA in neonatal myocardial cells treated with Pax8/LV7and LV7 for three consecutive days. (**D**) AnnexinV‐FITC/PI double staining detecting the apoptotic ratio after serum‐free medium for 24 hrs. LV7 was the vector of Pax8 lentiviral vector. The apoptotic rate of treated cells decreased compared with the control cells. Data are presented as mean ± S.D. **, *P* < 0.01, ***, *P* < 0.001 (magnification: ×100).

We performed experiments to determine whether Pax8 affects apoptosis of neonatal cardiomyocytes in the cultures deprived of serum for 24 hrs. Flow cytometry demonstrated that after three consecutive days, cultured‐myocardial cells were beating synchronously, stretched out the antenna, and could be fused up to 80%. The beating myocytes were stained positively with specific α‐MHC antibody (Fig. 4A). After transfected with the lentivirus vector, almost 80% of the cells expressed RFP, which is encoded by Pax8/LV7 recombination vector (Fig. 4B). Real‐time PCR analysis indicated that Pax8 mRNA expression increased by 300‐fold in the cells (Fig. 4C). Paired box gene 8 overexpression had a significant anti‐apoptotic effect on cardiac myocytes treated with the lentiviral Pax8 expression vector for three consecutive days (Fig. 4D), compared with control cells went through serum starvation 20. Thus, Pax8 attenuated excessive apoptosis in apoptosis model of cardiomyocytes.

### Differential expression of senescence markers in Pax8 knockout heart

**Figure 5:**
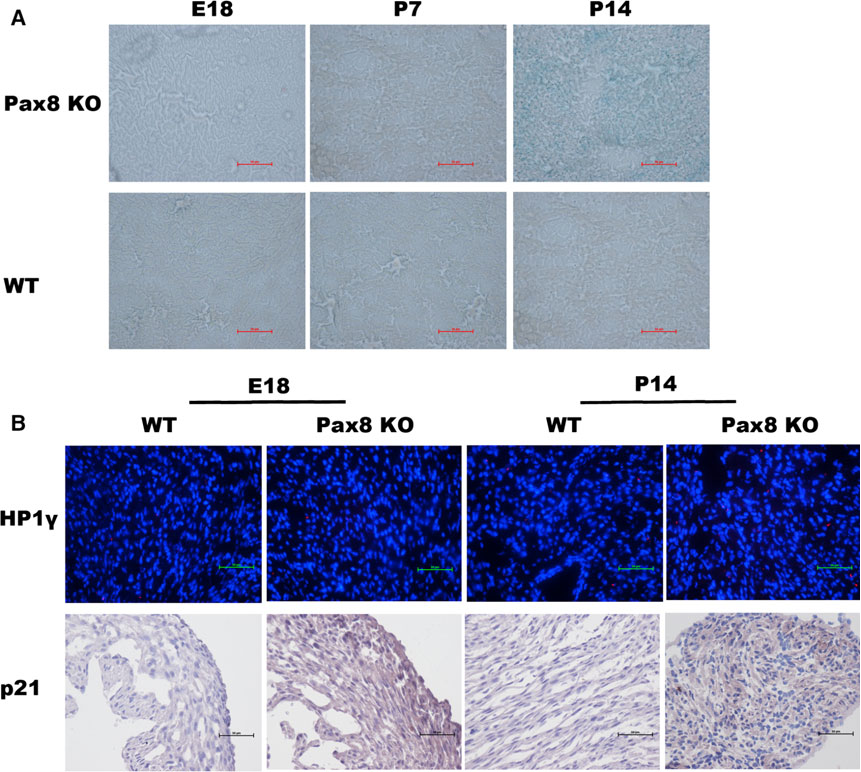
Senescence‐associated staining in heart. (**A**) SA‐β‐gal staining inPax8 null mice hearts at P14 stained with SA‐β‐gal signals (blue) spread to cardiomyocytes, whereas rare positive staining was found in E18, P7 and wild‐type sections. (**B**) Mice hearts at E18 embryos and P14 of the indicated genotypes stained with HP1γ (red), counterstained with DAPI (blue). Immunohistochemistry staining for p21 with DAB staining.

**Figure 6:**
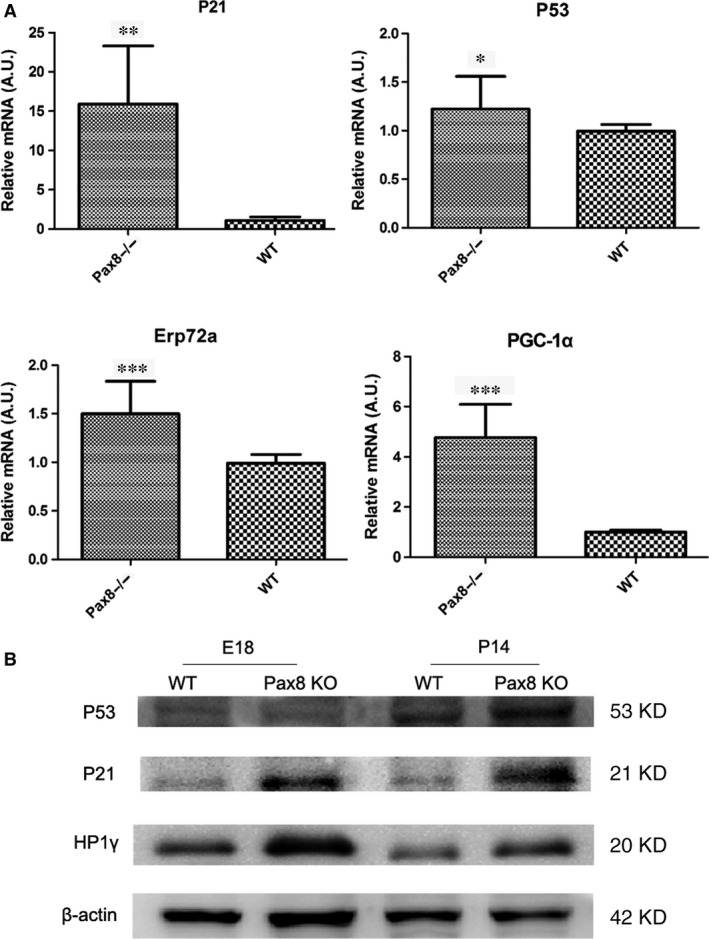
Quantified mRNA and protein level of senescence‐associated mediators. (**A**) The mRNA levels of p21, p53, PGC‐1α, and Erp72a by using quantitative RT‐PCR. (**B**) The bands of Western blot analysis of p21, p53 and HP1γ at E18 embryonic mice hearts and P14 mice hearts. Data are presented as mean ± S.D. *, *P* < 0.05. **, *P* < 0.01. ***, *P* < 0.001.

Immuno‐staining for the senescence marker SA‐β‐gal was conducted in Pax8 knockout hearts. The SA‐β‐gal signals were observed in sections of Pax8 null hearts at P14 (Fig. 5A). The blue SA‐β‐gal staining was seen only in senescent cells, which dispersed in myocardial cells. However, the E18, P7 Pax8 null mice heart and wild‐types rarely find SA‐β‐gal signals. To further document the senescence, we detected cyclin‐dependent kinase inhibitor and senescence‐associated heterochromatin. In case of p21, expression at P14 dramatically increased in Pax8 null hearts (Fig. 5B). Moreover, HP1γ is positive (red) in the nucleus in elder Pax8 null hearts compared with young E18 hearts and wild‐types (Fig. 5B). We also detected the mRNA and protein levels of the senescence mediators. The expression of p21 dramatically increased, with p53 weakly elevated in the hearts of the Pax8 mutant mice at P14. Collectively, mitochondria and endoplasmic reticulum‐associated PGC‐1α and Erp72a dramatically increased (Fig. 6A). The changes in protein levels of p21, and HP1γ significantly increased from E18 to P14, whereas p53 has a slight elevation in protein level (Fig. 6B).

In summary, we demonstrated the developmentally programmed senescence in Pax8 null heart, characterized by SA‐β‐gal, cyclin‐dependent kinase inhibitor, and senescence‐associated heterochromatin.

### ALK3 silencing induced senescence could be rescued by Pax8 expression in H9C2 cells

**Figure 7:**
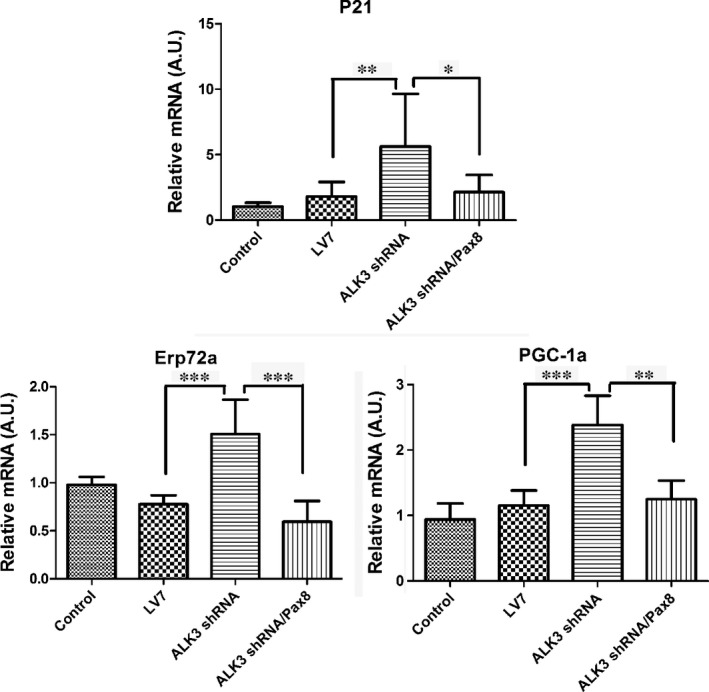
ALK3 and Pax8 pathway in the role of senescence in H9C2. LV7 was the vector of ALK3 shRNA and Pax8 overexpression vectors. The mRNA levels of cyclin‐associate mediators of p21, PGC‐1α, and Erp72a were determined by quantitative RT‐PCR. Data are presented as mean ± S.D. *, *P* < 0.05. **, *P* < 0.01. ***, *P* < 0.001.

The possible role of ALK3‐Pax8 pathway in cardiac cell senescence was explored by transfecting H9C2 cells with ALK3 shRNA and Pax8 expression lentiviral vectors. We observed that the mRNA levels of p21, PGC‐1α, and Erp72a increased when ALK3 was down‐regulated, and Pax8 slightly reversed ALK3‐induced senescence in mRNA level (Fig. 7). The protein level of p21 and HP1γ elevated with Pax8 down‐regulated and silencing of ALK3 in H9C2 cells (Fig. S2). The ALK3‐Pax8 pathway may regulate the cellular senescence during cardiac development.

## Discussion

Ventricular septal defect is a typical malformation in CHD. Septum dysplasia is complicated and sophisticated induced by genetic and environmental factors. In this study, we report that Pax8 is the possible downstream signalling molecule to ALK3 in heart development. Cardiac‐specific ALK3 deletion mice died in the middle embryonic stage and has VSD with endocardial cushion and trabecular muscles agenesia 9. The Paxs are a family of nine developmental related genes, these highly conserved genes are expressed at embryonic stage, whereas they are silent during terminal differentiation in most mature tissues or organs 21. Our previous study has found that Pax8 expression is down‐regulated by 7.1‐fold in cardiac‐specific ALK3^F/−^ mice 10, and the systemic Pax8^−/−^ mice died at about 20 days after birth, cardiac histological examination has shown the presence of VSD in embryonic stage 11. It is predicted that Pax8 may be one of the ALK3 downstream signalling molecules that have a pivotal function in the formation of the heart and other tissues during embryonic development. First, we verified the ALK3‐Pax8 pathway *in vitro*. Paired box gene 8 knockdown aggravated apoptosis, overexpression of Pax8 rescued apoptosis induced by ALK3 silencing in H9C2 cells, and its anti‐apoptotic function was seen in primary cardiomyocytes. Second, we further explored the function of Pax8 in senescence.

Bone morphogenetic protein signalling is largely activated through its receptors, BMP type II receptor and ALK. The classical downstream signalling of ALK include Smad and non‐Smad pathways 22, which are critical in regulation of cell apoptosis and proliferation. Phosphorylation of Smad1/5/8 and nucleus translocation with Smad4 activates the BMP signalling. The relationship between Smad and Pax8 still remain unknown. We have not found the elevation of phosphorylated Smad1/5/8 with Pax8 silencing in H9C2 cells (Fig. S3), implying that Pax8 may participate in apoptotic regulation in non‐Smad pathway or it may be a downstream molecular of Smad in ALK3 pathway.

Ventricular septum development is closely related to the coordination of gene networks, such as Nkx2.5, Tbx and GATA family 23, 24, 25. No rounded system could illuminate the potential mechanism of septum morphogenesis. Apoptosis and proliferations are important molecular mechanism involved in heart development. Nowadays, senescence may be a possible mechanism in embryonic development, which is the developmentally programmed senescence 14, 15. It is a new perspective in development areas of vertebrate, whereas senescence is, in general, related to aging and most pathological damage.

We wondered if cellular senescence mediates heart development by Pax8. Therefore, we compared Pax8 null mice with wild‐type, E18 embryonic hearts, P7 hearts at postnatal 7 days and P14 hearts at postnatal 14 days. SA‐β‐gal staining is a classical sign in measurement of senescence. In this study, SA‐β‐gal activity increased in Pax8 null P14 ones, which was only stained senescent cells. We can see rarely positive staining in younger Pax8 null mice hearts before 14 days and wild‐types. The senescence‐associated heterochromatin markers, such as HP1γ, have a remarkable concentration in elder Pax8 null mice hearts. It is a pity that we did not collect embryos before E18 because of the limited fertility of Pax8 null mice, we will sequentially collected embryos in future. In support of this, we detected cyclin‐dependent kinase inhibitors p21, p53, p19, p16 and other senescence‐associated mediators such as mitochondria‐associated PGC‐1α, cytochrome c, endoplasmic reticulum‐associated (ER‐associated), Bip and XBP1. We found that the expression of p21 was more prominent in Pax8 null old mice than the other mediators, and p19 and p16 were not significantly affected, suggesting that Pax8 knockout induced senescence because of p53/p21 pathway, rather than an increase in p16 and p19. Moreover, PGC‐1α and Erp71a had a remarkable increase in Pax8 null ones, which implied that ER stress and mitochondria metabolism may mediate developmental senescence. As we has partially verified the relationship between ALK3 and Pax8 in apoptosis, next, we observed that ALK3 silencing induced p21 increase in H9C2 cells, while the protein level of p21 and HP1γ elevated in Pax8 knockdown group. As we are establishing the Pax8 transgenic mice, more function and mechanism in senescence between ALK3 and Pax8 in heart development should be investigated in the future. In summary, our data suggest that Pax8 may take part in the development of heart dysplasia, and Pax8 deficiency aggravated programmed senescence and apoptosis.

The developmental senescence may participate in organogenesis of Pax8 null mice heart. We found that p21 but not p16 and p19 may serve as a critical molecule involved in developmental senescence. The mechanism of p21‐mediated developmentally programmed senescence through TGF‐β‐SMAD and PI3K‐forkhead box protein O pathways and pregnant mice treated with TGF‐β pathway inhibitor (ALK5) decreased the SA‐β‐gal activity and p21 14. The expression of p21 is regulated through p53‐dependent and p53‐independent pathways 26. Our data may indicate, at least in part, that ALK3 pathway may also partially mediate p21 involved in developmental senescence.

Developmental senescence has been seen in human, quail and chicken embryos, suggesting that this process is a conserved process of program development among vertebrates. Physiological aging and apoptosis can be seen in mammalian development. As an important physiological process in organogenesis, increased apoptosis is involved in the development of the atrioventricular cushions, septum, out‐flow tract, arterial valves, and ventricular papillary muscles 27, 28, 29. Although many studies suggest that senescent cells are resistant to apoptosis 30, 31, aging associated disruption result in increased susceptibility to apoptosis in some cells and decreased apoptosis in other cells 32, 33, its largely depend on the cell type and damage intensity. Senescence‐associated apoptosis includes caspase‐dependent and caspase‐independent apoptosis 34. These apoptosis are the most important way to eliminate unwanted cells. Inhibition of the apoptotic program causes morphological defects, indicating the existence of compensatory mechanisms 35. Failure to experience senescence activates a compensatory apoptotic pathway. A key molecule in both processes is the p53. The apoptotic program is induced by p53 of pro‐apoptotic proteins which triggers the caspase cascade, while senescence is driven by activation of p53/p21/Rb pathway. Apoptosis and senescence are interconnected, enabling their complementation during development.

Taken together, we conclude that the Pax8 functions as a downstream molecule for regulation of cardiac‐specific ALK3, a major element of the BMP signalling pathway, by which Pax8 knockout leads to the heart dysplasia as a result of excessive senescence and apoptosis in cardiac myocytes. Our findings also reveal that specific intervention targeting at Pax8 and ALK3 to inhibit apoptosis and senescence may become a novel strategy in the prevention and treatment of heart dysplasia.

## Conflicts of interest

The authors confirm that they have no conflicts of interest.

## Supporting information


**Figure S1** The apoptotic effect of Pax8 knockdown evoked by ALK3 silencing in H9C2 cells.

**Figure S2** Expression of senescence‐associated molecules in H9C2 cells.

**Figure S3** Expression of phosphorylated‐Smad 1/5/8 protein with Pax8 silencing in H9C2 cells.

**Table S1** Quantitative RT‐PCR primers.
**Table S2** The target sequence for Pax8.
