# Supplementary figures and images for: Pax8 plays a pivotal role in regulation of cardiomyocyte growth and senescence
Source: J Cell Mol Med. 2016 Jan 19;20(4):644–54. doi: 10.1111/jcmm.12779 (PMC5125375; doi:10.1111/jcmm.12779)

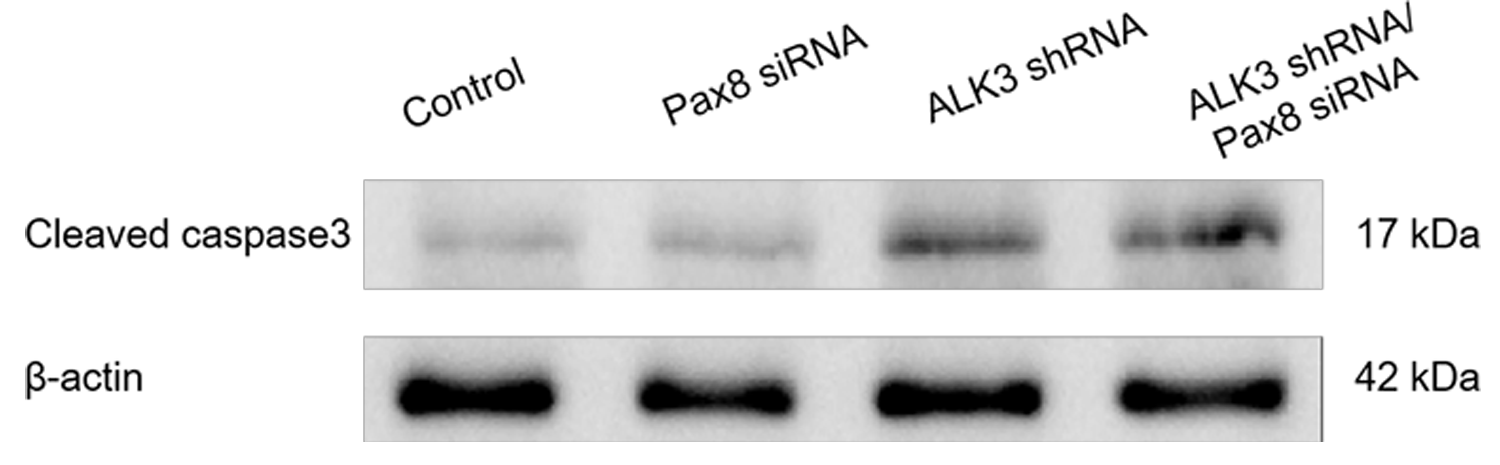

Supplement: Supplementary file 1 — Figure S1 The apoptotic effect of Pax8 knockdown evoked by ALK3 silencing in H9C2 cells. [file JCMM-20-644-s001.tif]

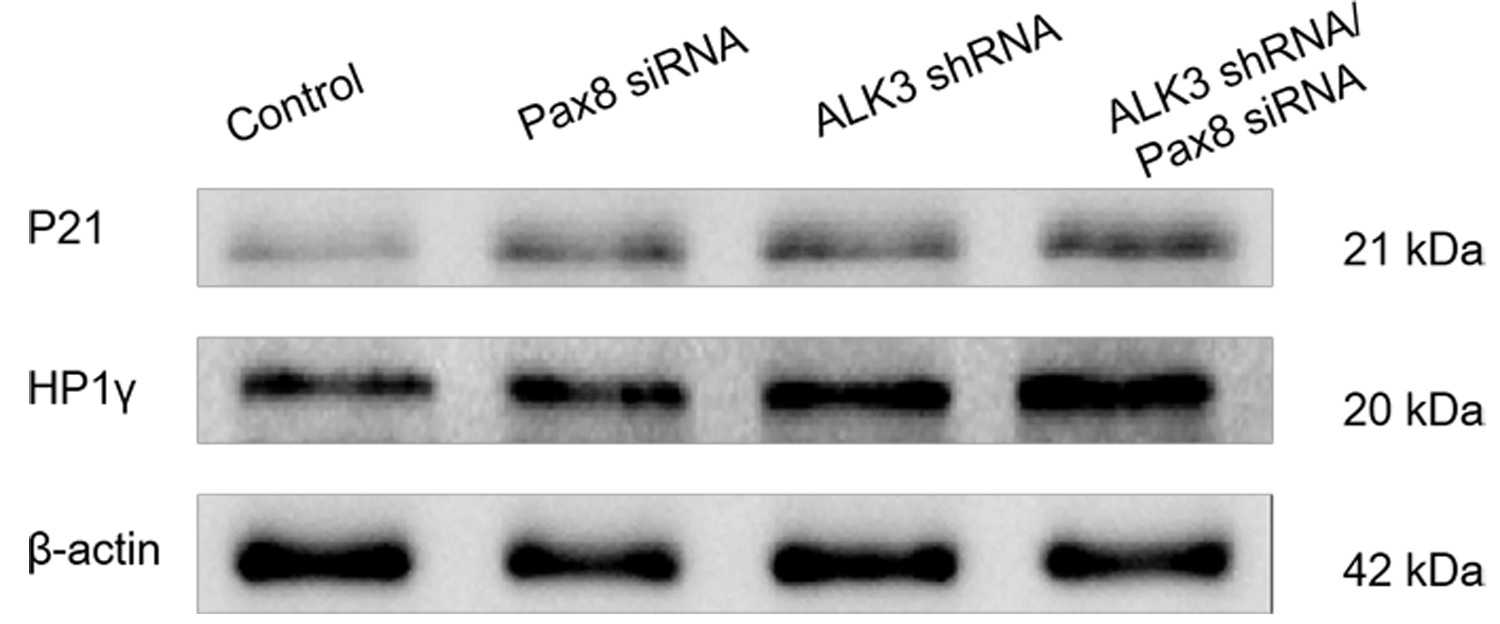

Supplement: Supplementary file 2 — Figure S2 Expression of senescence‐associated molecules in H9C2 cells. [file JCMM-20-644-s002.tif]

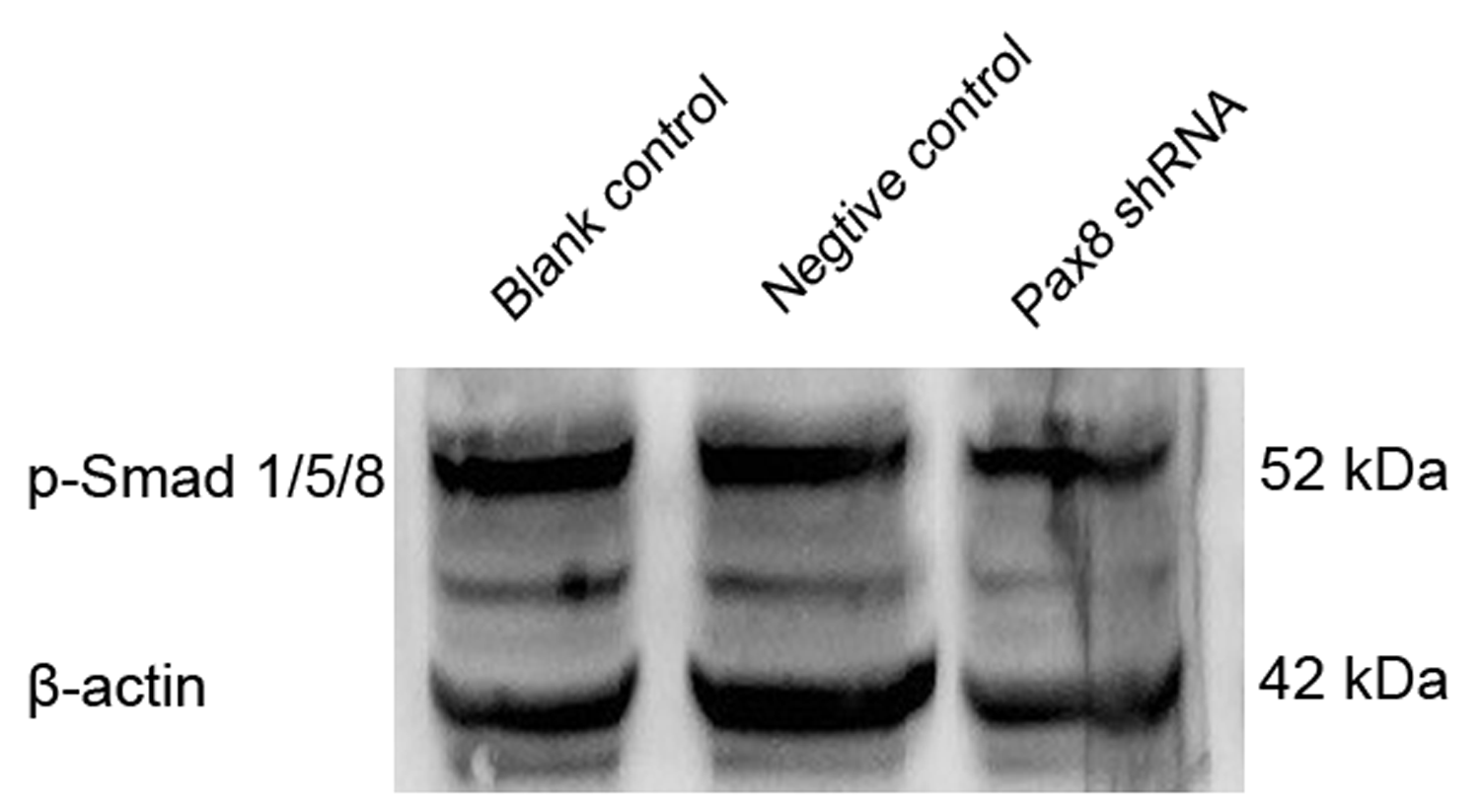

Supplement: Supplementary file 3 — Figure S3 Expression of phosphorylated‐Smad 1/5/8 protein with Pax8 silencing in H9C2 cells. [file JCMM-20-644-s003.tif]
