# Supplementary material for: Pax8 plays a pivotal role in regulation of cardiomyocyte growth and senescence
Source: J Cell Mol Med. 2016 Jan 19;20(4):644–54. doi: 10.1111/jcmm.12779 (PMC5125375; doi:10.1111/jcmm.12779)
Supplement: Supplementary file 4 — Table S1 Quantitative RT‐PCR primers. Table S2 The target sequence for Pax8. [file JCMM-20-644-s004.docx]

**Supplement Table 1 quantitative RT-PCR primers**

**Name Sequence**

**Pax8:**  F: 5-ATGCCTCACAACTCGATCAGA-3;

R: 5-ACAATGCGTTGACGTACAACTT-3;

**GAPDH**: F: 5- AGGTCGGTGTGAACGGATTTG -3,

R: 5- TGTAGACCATGTAGTTGAGGTCA -3.

**P21**: F: 5’-GGACGTCCCACTTTGCCAGCAG-3’

R: 5’-GAGCGCATCGCAATCACGGC-3’

**PGC1a**: F: 5’-TCGGGAGCTGGATGGCTTGGGA-3’

R: 5’-ACCAACCAGAGCAGCACACTCTA-3’

**P53:** F: 5’-GGCCCAAGTGAAGCCCTCCG-3’

R: 5’-GCCCAGGGGTCTCGGTGACA-3’

**Erp72a** F: TCCCATTGCTGTAGCGAAGAT

R: GGGGTAGCCACTCACATCAAAT

**Supplemental Table 2 The target sequence for Pax8**

| **Name sequence** |
| --- |
| **Pax8 siRNA** sense 5'-GGAAGUGAGUAUUCUGGCATT-3'  anti-sense 5'-UGCCAGAAUACUCACUUCCTG-3'  **Negative control** sense 5'-UACUCCGAACGUCCACGUTT-3'  anti-sense 5'-UGUACACGACGGAGCAGTT-3 |
